# Long-term and large-scale spatiotemporal patterns of soundscape in a tropical habitat of the Indo-Pacific humpback dolphin (*Sousa chinensis*)

**DOI:** 10.1371/journal.pone.0236938

**Published:** 2020-08-12

**Authors:** Wanxue Xu, Lijun Dong, Francesco Caruso, Zining Gong, Songhai Li

**Affiliations:** 1 Marine Mammal and Marine Bioacoustics Laboratory, Institute of Deep-sea Science and Engineering, Chinese Academy of Sciences, Sanya, China; 2 University of Chinese Academy of Sciences, Beijing, China; 3 Tropical Marine Science Institute, National University of Singapore, Singapore, Singapore; Tanzania Fisheries Research Institute, UNITED REPUBLIC OF TANZANIA

## Abstract

Little is known about the characteristics of ambient sound in shallow waters southwest of Hainan Island, China, a tropical habitat of the Indo-Pacific humpback dolphin. The spatiotemporal patterns of soundscape in this area were thus studied and described here. Acoustic data collected from February 2018 to February 2019 at ten monitoring sites, spanning ~200 km of the coastline, were analyzed. The ambient sound characteristics in the investigated area showed significant spatiotemporal variations. Sound levels centered at 0.5 and 1 kHz were higher during dusk and night than other times of the day at all monitoring sites except for one. Higher sound levels at frequencies above 8 kHz were documented during autumn and winter at all sites except for three of them. Biological and anthropogenic sound sources including soniferous fishes, snapping shrimps, dolphins, ships, pile-driving activities, and explosions were identified during spectrogram analyses of a subsample of the dataset. The shipping noise was frequently detected throughout the monitoring sites. Spatiotemporal variations of the soundscape in the investigated waters provided baseline information on the local marine environment, which will be beneficial to the protection of the vulnerable Indo-Pacific humpback dolphin population recently discovered in the investigated waters.

## Introduction

The soundscape of an environment is the aggregation of geophysical, biological and anthropogenic sounds present in it [1]. The research on marine soundscape could provide insights into the species composition of a given marine environment, provide valuable information on habitat quality, and reflect long-term changes of species distribution, biodiversity and human activities [2, 3].

In shallow waters, natural phenomena such as waves, wind and rain [4, 5] generate part of the ambient sound by interacting with the surface layer of the ocean [6, 7]. The wind contribution dominates from a few Hz to 30 kHz [4], surface waves cause mostly infrasonic sounds at frequencies from 10 to 100 Hz [4], and rainfall also produces energy peaks from 15–20 kHz [8]. Biological sounds are related to marine mammals [9, 10], fishes [11, 12] and invertebrates [13], and their activities [14]. Marine mammals produce various types of sounds as efficient mean to exchange information with conspecific and interact with the surrounding environment, using signals at various frequency bandwidths [10, 15]. For example, toothed whales emit echolocation clicks when locating prey and narrow-banded frequency modulated whistles when socializing [10]. In contrast, some baleen whales emit songs which are generally produced at low frequencies and consist of a combination of pulses and tonal sounds [15]. Soniferous fishes produce impulsive or frequency-modulated sounds below 10 kHz with different types of signals and biological activities [16], while most fish calls at low-median frequencies (<2kHz) [16, 17]. They use sounds to perceive the environment and to coordinate certain behaviors such as reproduction or territory defense [17]. Reef fishes use sounds to guide settlement onto reefs [18]. The key biological sound sources in shallow waters are snapping shrimps, especially in subtropical and tropical areas [13, 19, 20]. They emit broadband pulse signals (up to 200 kHz) [21], which are affected by time of day [22], environmental variables [23] and social context [24]. Higher rate of snaps were usually recorded during dusk and night for individuals and same-sex pairs, while different daily trends for opposite-sex pairs and a mixed-sex group [24, 25]. Anthropogenic sources are generally marine traffic, geophysical explorations, wind farm constructions, pile-driving, military exercises and other noisy human activities [26, 27]. In general, ship noise is believed to be the main contributor to man-made sounds in shallow waters throughout the world [14, 28, 29], particularly at low frequencies (< 1 kHz) [30].

Man-made underwater sound levels have increased at a rate of 3 dB per decade [31, 32]. This increase is mainly associated to the growing shipping activity, and global concern is concurrently growing up about the impact of noise on marine life [33]. For marine mammals, anthropogenic sounds may cause behavioral alterations, including: changes in surfacing and diving patterns [34], stranding [35], hearing loss [36, 37], and communication masking [26, 38]. For example, in shallow waters, small vessels traveling at 5 knots can reduce the communication range of bottlenose dolphins by 26% within 50 m [39]. The rates and signal characteristics of dolphin sounds seem to be affected by man-made noise as well [40–42]. Understanding the effect of noise on dolphin population can improve the effectiveness of protection actions, especially for vulnerable and endangered species.

The waters southwest of Hainan Island, China, are a tropical habitat of Indo-Pacific humpback dolphin (IPHD, *Sousa chinensis*), which was only recently discovered [43]. IPHD mainly lives in shallow and coastal waters [44, 45], where they are overlap with human activities that generate underwater noise [46, 47]. The species has already been classified as “Vulnerable” by the International Union for Conservation of Nature (the IUCN Red List of Threatened Species 2018). Recently, PAM studies reported that distribution, acoustic behavior, and habitat use of IPHD in this area are strongly influenced by the abundance of soniferous fishes and the species seemed to be more acoustically active in locations with lower levels of noise [47, 48]. Research on the log-term and large-scale variation of soundscape in Hainan waters would help to better understand the marine acoustic environment and eventually benefit the conservation of IPHD. The soundscape of other two IPHD habitats located in subtropical areas have been previously studied [49, 50]. In two sites of waters off Taiwan, China, two frequency bands (150–300 Hz and 1.2–2.4 kHz) were studied during a 3-month monitoring, and it was reported that the lower frequency band was mainly associated with container vessels at one site, while the higher frequency band was from fish chorus at nighttime in both sites [49]. The variation of soundscape in the Pearl River Estuary waters was studied during a 16-day monitoring and reported a significant variation in diel and tidal soundscapes, but with the majority of IPHD sounds detected during periods without fish sounds [48]. However, in that specific study the acoustic monitoring was conducted during a very short monitoring period.

Although these studies provided important regional information about the acoustic space occupied by IPHD, a long-term and large-scale spatiotemporal analysis is needed to generate a comprehensive understanding of its habitat [47, 48]. Different waters have different soundscape signatures related to distinctive physical and biological features [49, 51]. For example, seabed hardness and snap rate have a significant relationship that may be accounted for by the higher reflection properties of hard surfaces compared to soft substrates [52]. The IPHD habitat quality in the waters southwest of Hainan Island and potential impacts of anthropogenic activities on this habitat remain unclear. Underwater soundscape is considered a reliable index to indicate habitat quality [53], characterize sound sources in a specific underwater environment, and evaluate potential impacts of anthropogenic activities [54].

In this study, we aimed to describe how the soundscape varies during long-term monitoring and at a large spatial scale (~200 km along the coast) in an important IPHD habitat. Specifically, we set eleven monitoring sites in waters southwest of Hainan Island and adopted stationary passive acoustic monitoring (PAM) platforms to collect one-year data. Then, the temporal and spatial patterns of soundscape in frequency range between 20–144 kHz were analyzed. The possible effect of different seabed features on the soundscape were also studied, and the main biological and anthropogenic sound sources were described in this tropical habitat of a vulnerable coastal dolphin species.

## Methods

### Ethics statement

This study was performed under an Ethical Statement from the Institute of Deep-sea Science and Engineering, Chinese Academy of Sciences, with the number of IDSSE-SYLL-MMMBL-01.

### Study area and PAM sites

**Fig 1:**
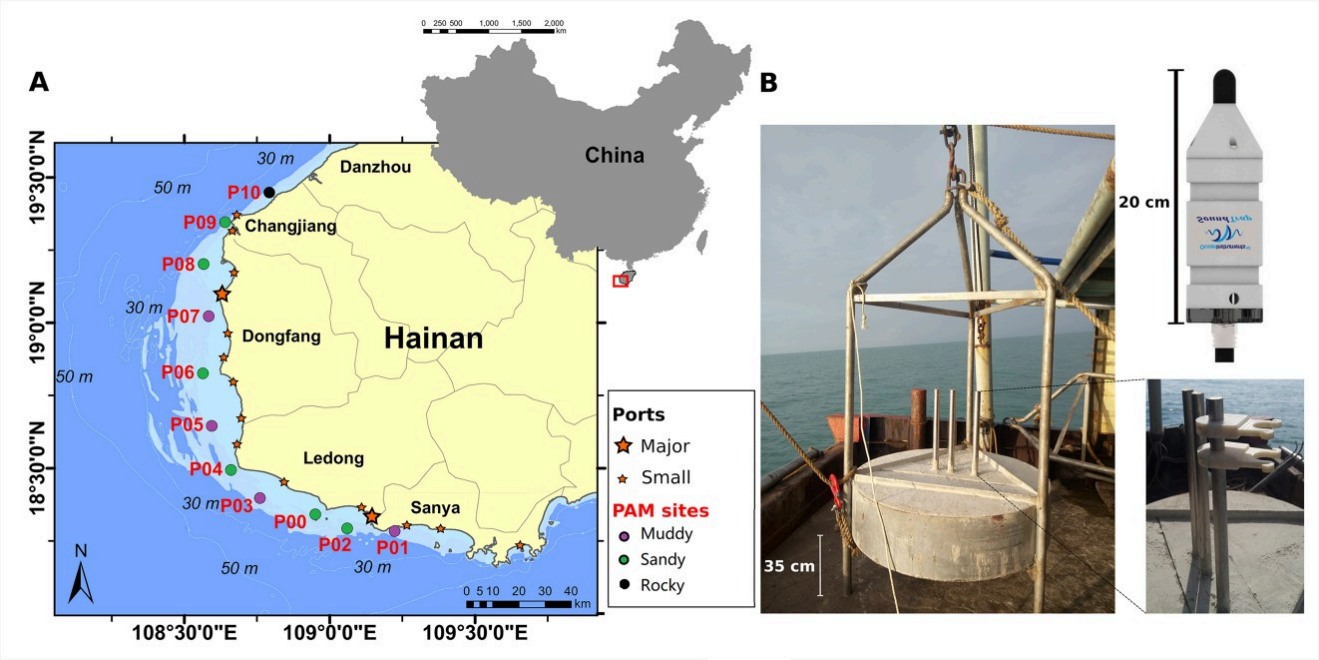
Map of the study area and monitoring sites (A) and picture of the acoustic platform (B). Monitoring sites are marked with ○, and different colors means different types of seabed. The major ports and other ports are marked with ☆. The SoundTrap recorder was fixed on a stainless steel column at the center of each platform.

Fig 1A shows the location of eleven PAM sites in the study area. Six stations were located in sandy seabed, four in muddy bottom, and one in a rocky area (Table 1).

**Table 1:** Information about each PAM site (Latitude, Longitude, depth, and bottom).

Monitoring site	Latitude	Longitude	Depth (m)	Bottom
P00	18°20.567′	108°57.068′	10	sandy
P01	18°17.091′	109°13.442′	15.5	muddy
P02	18^o^17.662′	109^o^ 03.636′	18	sandy
P03	18°23.911′	108°45.624′	13.7	muddy
P04	18°29.705′	108°39.678′	16.3	sandy
P05	18°38.794′	108°35.714′	10.2	muddy
P06	18°49.652′	108°33.854′	14	sandy
P07	19°00.358′	108°35.603′	11.8	muddy
P08	19°11.016′	108°34.362′	10.5	sandy
P09	19°20.462′	108°39.599′	14.3	sandy
P10	19°26.171′	108°47.958′	15	rocky

Each platform, composed of a concrete structure and exposed stainless steel frame, has a total weight of about two tons and was placed on the seabed (Fig 1B). The stainless steel frame was used to protect the acoustic recorder. The distance between adjacent monitoring sites was set to be about 20 km to obtain distinct soundscape features from each site and also to cover all the potential IPHD habitat.

### Acoustic data collection

An acoustic recorder (SoundTrap 300HF, Ocean Instruments, New Zealand) was fixed on one of the short stainless steel columns at the center of each platform (Fig 1B). The SoundTrap presented a sensitivity of -203 ± 3 dB re V/μPa within a frequency range of 20 Hz-150 kHz and it was set to have a maximum SPL before clipping of 172 dB re 1μPa. The recorder acquired data at a resolution of 16 bits and a sampling rate of 288 kHz. In order to balance the battery life and data storage, a duty cycle of 5 minutes in every 30 minutes was used. Each 5-minute continuous acoustic data were stored as a single audio file. In this way, each acoustic recorder was able to keep acquiring data for about 60 days. Therefore, the SoundTrap on each platform was regularly retrieved and redeployed by professional divers approximately every two months. Unfortunately, the platform deployed at site P02 was lost after the first deployment. Therefore, there were no data acquired from site P02 in this study. Recorders of sites P00-P06 were first deployed on February 2018. Recorders of sites P07, P09 and P10 were first deployed on May 2018, and recorder of site P08 were first deployed on September 2018.

### Soundscape analysis

All acoustic data were analyzed in MATLAB (Version R2017a, The MathWorks Inc.). In order to reveal the variation patterns of ambient sound during the investigated period, broadband sound pressure levels (broadband SPLs) and octave band sound pressure levels (octave band SPLs) were calculated. Broadband SPLs were used to show the change of SPLs in the total frequency band of acquisition. For each audio file (5 minutes), the root-mean-square broadband SPL in the frequency range between 20 Hz and 144 kHz for every 1-min signal segment was calculated. Such frequency band was determined based on the frequency response (0.02–150 kHz) and sampling rate (288 kHz) of the recording system. The root-mean-square octave band SPLs for 11 octaves including 62.5 Hz (44–88 Hz), 125 Hz (88–177 Hz), 250 Hz (177–355 Hz), 500 Hz (355–710 Hz), 1000 Hz (710–1420 Hz), 2000 Hz (1420–2840 Hz), 4000 Hz (2840–5680 Hz), 8000 Hz (5680–11360 Hz), 16000 Hz (11360–22720 Hz), 32000 Hz (22720–45440 Hz), and 64000 Hz (45440–90880 Hz) in every 1-min signal segment were also calculated.

The datasets of broadband SPLs and octave band SPLs were tested for normality with a Kolmogorov–Smirnov test. When datasets were not normally distributed (p<0.05), Kruskal-Wallis test and Mann-Whitney U test were used in this study. Spatial comparisons of broadband SPLs and octave band SPLs were made among all sites. Features of sea bottom and water depth were considered environmental factors that can affect the ambient noise [55]. In this study, the depth of the recorders at the PAM sites were between 10 and 16 m and considered to be comparable among each other. However, the monitoring sites had different sea bottom features, with P00, P04, P06, P08 and P09 characterized by sandy seabed, P01, P03, P05 and P07 characterized by muddy floor, and P10 characterized by a rocky environment. Therefore, broadband SPLs and octave band SPLs were assigned to different sea bottom categories, and the variation of ambient sound levels within each benthic environment was analyzed. Kruskal-Wallis test was conducted to explore whether the characteristic of seabed had effects on the acoustic signature of local soundscape and Mann-Whitney U test was conducted to explore the difference of acoustic signature between different sea floor features.

For each site, temporal trends of soundscape were determined by comparing broadband SPLs and octave band SPLs among different time of day (dawn, day, dusk and night) and among seasons (spring, summer, autumn, and winter). Dawn and dusk were defined respectively as the periods ±90 min from sunrise and sunset. Day was defined as the period from -90 min of sunrise to +90 min of sunset, and night was defined as the period from -90 min of sunset to +90 min of sunrise. The sunrise and sunset times were obtained from Time and Date AS website (http://www.timeanddate.com). Seasons were defined as follows: spring (March-May), summer (June-August), autumn (September-November) and winter (December-February). The average broadband SPLs and octave band SPLs for different time of day and for each season of the year were calculated from the data. Kruskal-Wallis and Mann-Whitney U tests were used to assess the significance of differences of broadband SPLs and octave band SPLs among time of day (dawn, day, dusk and night) and between different time of day at each site. Kruskal-Wallis test was conducted to examine the difference of acoustic characteristics among seasons (spring, summer, autumn and winter), which was also assessed by Mann-Whitney U test. In this study, all statistical analyses were performed by using IBM SPSS Statistics 20.0 (IBM Corp., Armonk, NY, USA).

### Description of the main biological and anthropogenic sound sources

A subsample of the large dataset was analyzed to describe the main biological and anthropogenic sound sources recorded in coastal waters southwest of Hainan Island during this long-term and large-scale PAM study. Spectrograms of three-day sound recordings during the new moon period were manually inspected (both aurally and visually) for all the monitoring sites by using the CHORUS toolbox [56]. The reasons justified the application of this data analysis protocol were: (1) the manual examination of all the dataset was a big time-consuming effort due to the large amount of recordings acquired; (2) in coastal waters, an higher occurrence of biological sounds was commonly documented during the new moon phase [57, 58]. The three days for manual examination were selected one day before to after the new moon day. Information about the new moon days in the study area were obtained from Time and Date AS website (http://www.timeanddate.com). Each spectrogram of the three-day acoustic data was created by two steps: (1) data were first Fourier transformed in 1-s windows in MATLAB, and power spectral densities (PSDs) of each 1-s window were produced as pre-processed data; (2) the CHORUS GUI toolbox was used to plot pre-processed data to make a three-day spectrogram for visual and aural examination of the main biological and anthropogenic sound sources. Following a recent investigation on the acoustic habitat variation of IPHD in part of the study area [47] and similar studies on different geographical areas [50, 59–61], the occurrence of sounds from soniferous fishes and ships were inspected in detail. Fish calls were considered as a possible prey distribution factor for IPHD, and ship noise was described as the main chronic man-made sound influencing its habitat. Therefore, information about occurrence of fish calls and ship noise were reported during the spectrogram inspection of the dataset subsample.

## Results

A total of 122,832 five-minute files of acoustic data were collected during the investigated period (from February 2018 to February 2019), at ten PAM sites (P00, P01, P03, P04, P05, P06, P07, P08, P09, and P10) in shallow waters southwest of Hainan Island (Table 2). The monitoring activity started a few months late at sites P07- P10, for that reason lack of data in these locations was reported at the beginning of the experiment. The other acquisition stops were mainly related to the battery duration of the SoundTrap. All data were analyzed for spatial and temporal soundscape patterns.

**Table 2:** Acoustic effort in number of days by month and deployment sites.

Year	Month	Monitoring site									
		P00	P01	P03	P04	P05	P06	P07	P08	P09	P10
2018	February	2	2	1	-	-	-	-	-	-	-
	March	31	31	31	31	29	29	-	-	-	-
	April	30	30	30	30	30	30	-	-	-	-
	May	10	8	11	9	16	8	3	-	3	4
	June	30	30	30	30	30	30	30	-	30	30
	July	27	27	27	29	27	31	31	-	31	28
	August	23	24	23	-	-	3	5	-	13	-
	September	30	30	30	4	3	20	19	17	30	3
	October	9	9	12	31	31	31	31	31	22	31
	November	16	17	17	30	30	15	30	28	14	30
	December	31	31	31	31	31	4	31	31	31	31
2019	January	24	29	19	31	24	31	28	12	27	22
	February	28	28	-	28	28	28	28	-	28	28
day	-	291	296	262	284	279	260	236	119	229	207

### Spatial patterns

**Fig 2:**
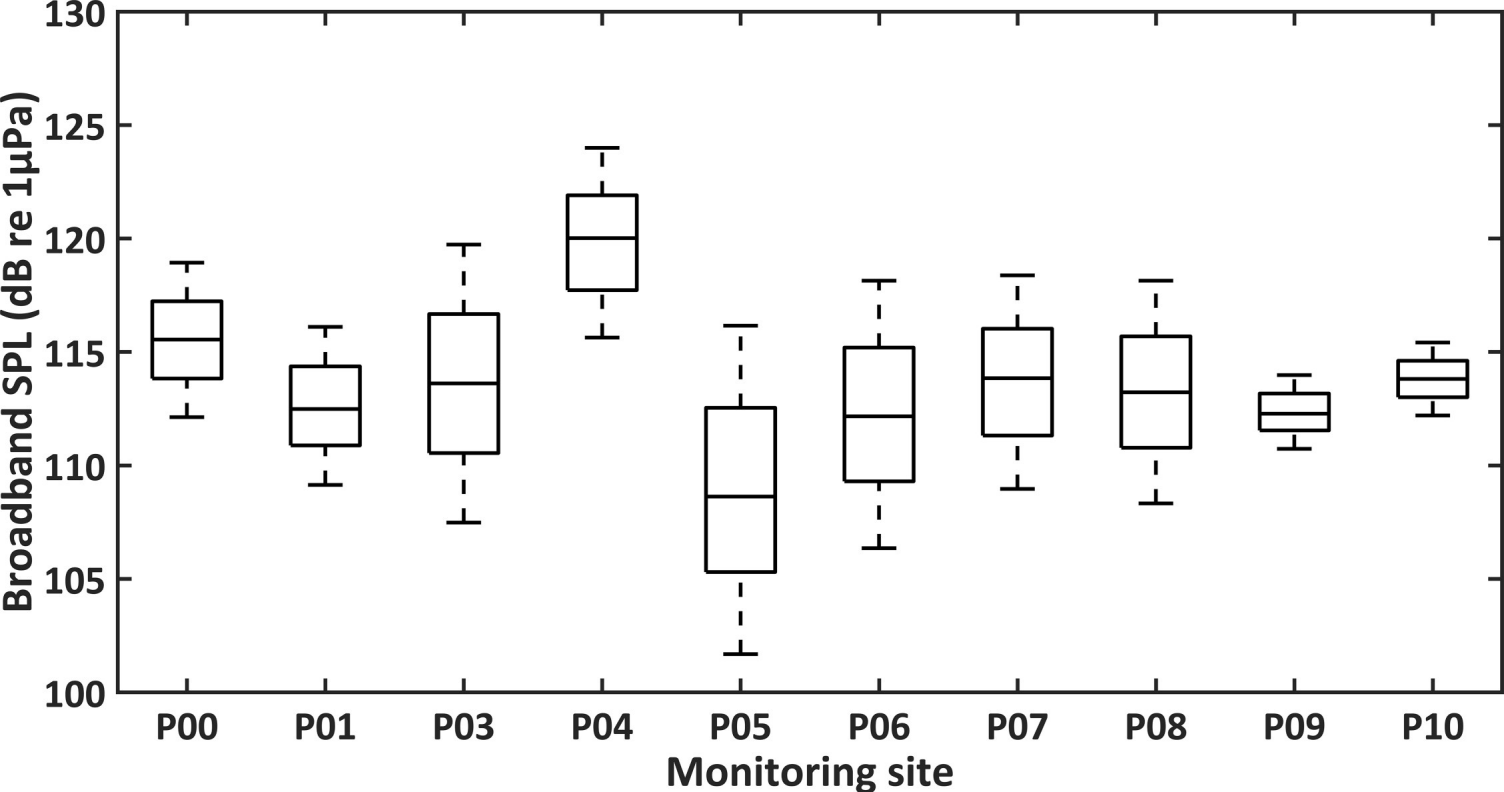
Spatial variation of broadband sound pressure levels (broadband SPLs) at the PAM sites. The differences of broadband SPLs from 20 Hz to 144 kHz among ten monitoring sites (Median; Whisker: 25^th^–75^th^).

**Fig 3:**
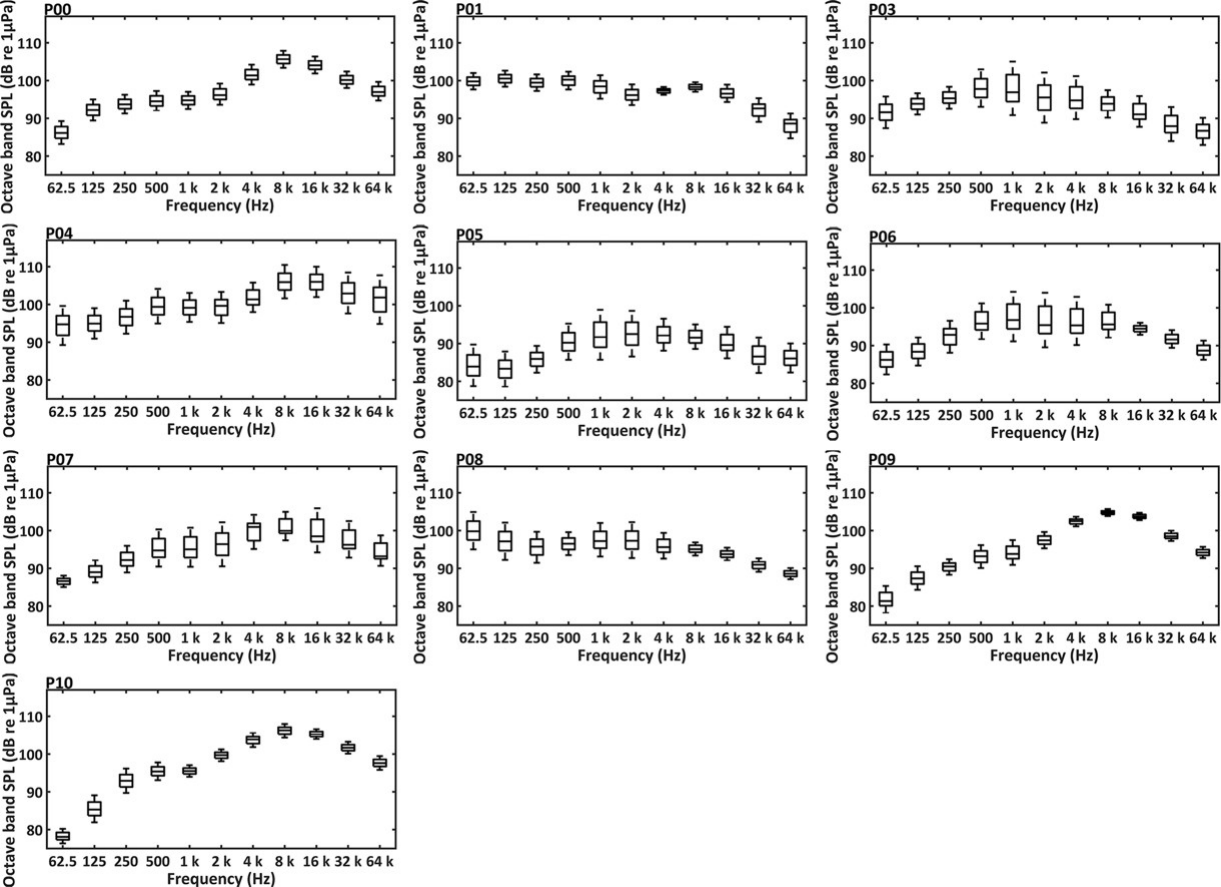
Spatial variation of octave band sound pressure levels (octave band SPLs) at different frequencies at the PAM sites. The differences of eleven octave band SPLs among ten monitoring sites (Median; Whisker: 25^th^–75^th^).

Average broadband SPLs for the ten sites P00, P01, P03, P04, P05, P06, P07, P08, P09, and P10 were 115.7, 113.3, 113.9, 119.7, 112.6, 113.3, 114.0, 113.7, 113.0, and 114.1 dB re 1μPa, respectively (S1 Table). Significant difference in broadband SPLs existed among the ten sites (Kruskal-Wallis test, df = 9, p < 0.001; Fig 3). P04 and P05 were found to have the highest and lowest average broadband SPLs, respectively (Kruskal-Wallis test, df = 9, p < 0.001; Mann-Whitney U test, df = 1, p < 0.001; Fig 2 and S1 Table).

The ten PAM sites were further compared in terms of octave band SPLs (Fig 3 and S2 Table). SPLs at certain bands were more variable than others. Octave band SPLs centered at 62.5 Hz was the lowest at site P10 (Mann-Whitney U test, df = 1, p < 0.001). Octave band SPLs centered at 16, 32, and 64 kHz were higher at sites P04 and P10 than that at other sites (Mann-Whitney U test, df = 1, p < 0.001). Octave band SPLs centered at 125, 250, and 500 Hz were the lowest at site P05 (Mann-Whitney U test, df = 1, p < 0.001).

**Fig 4:**
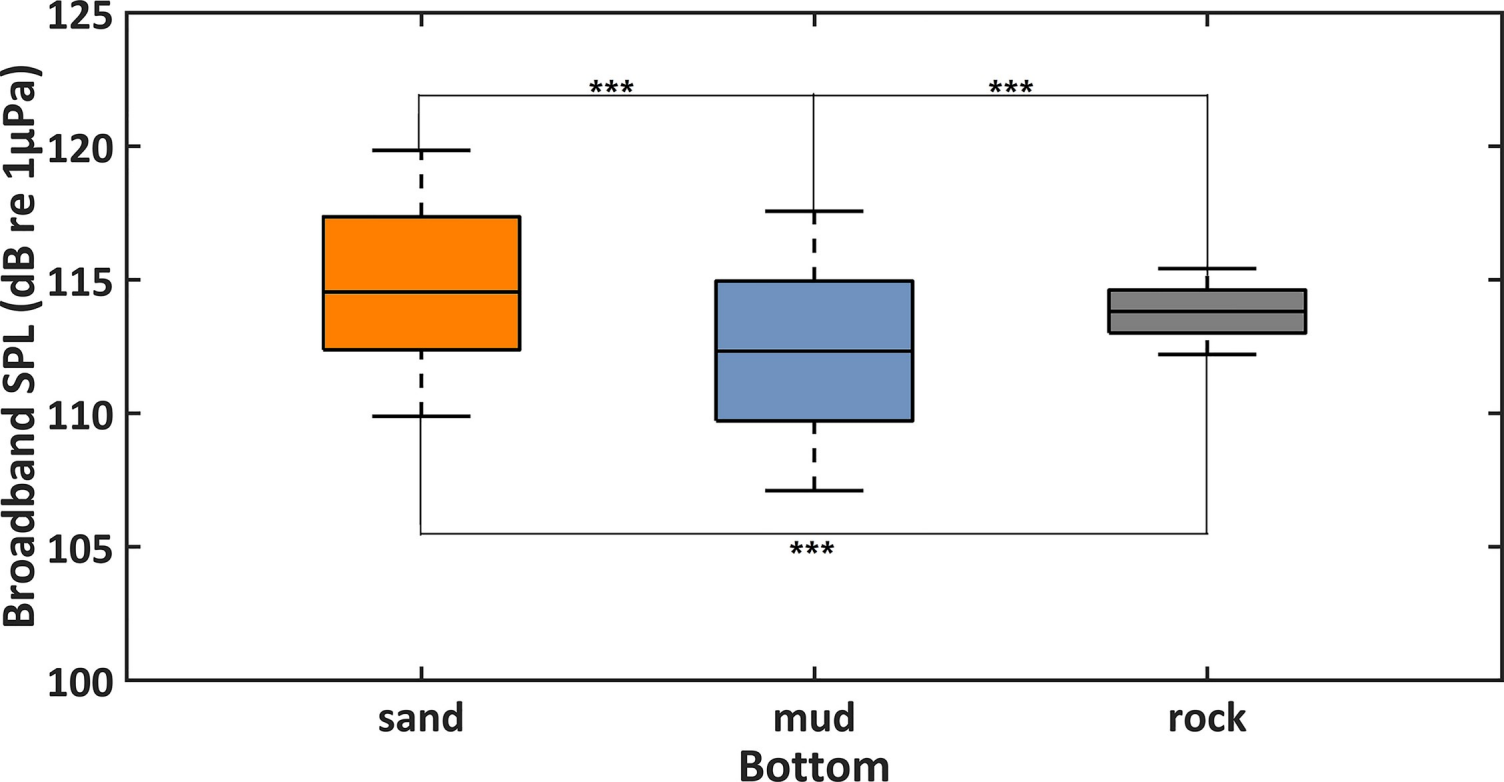
Comparison of broadband sound pressure levels (broadband SPLs) among different seabed environments. The three categories were muddy, sandy and rocky sea floors. Differences are marked with *** for p-level < 0.001 (Mann-Whitney U test).

**Fig 5:**
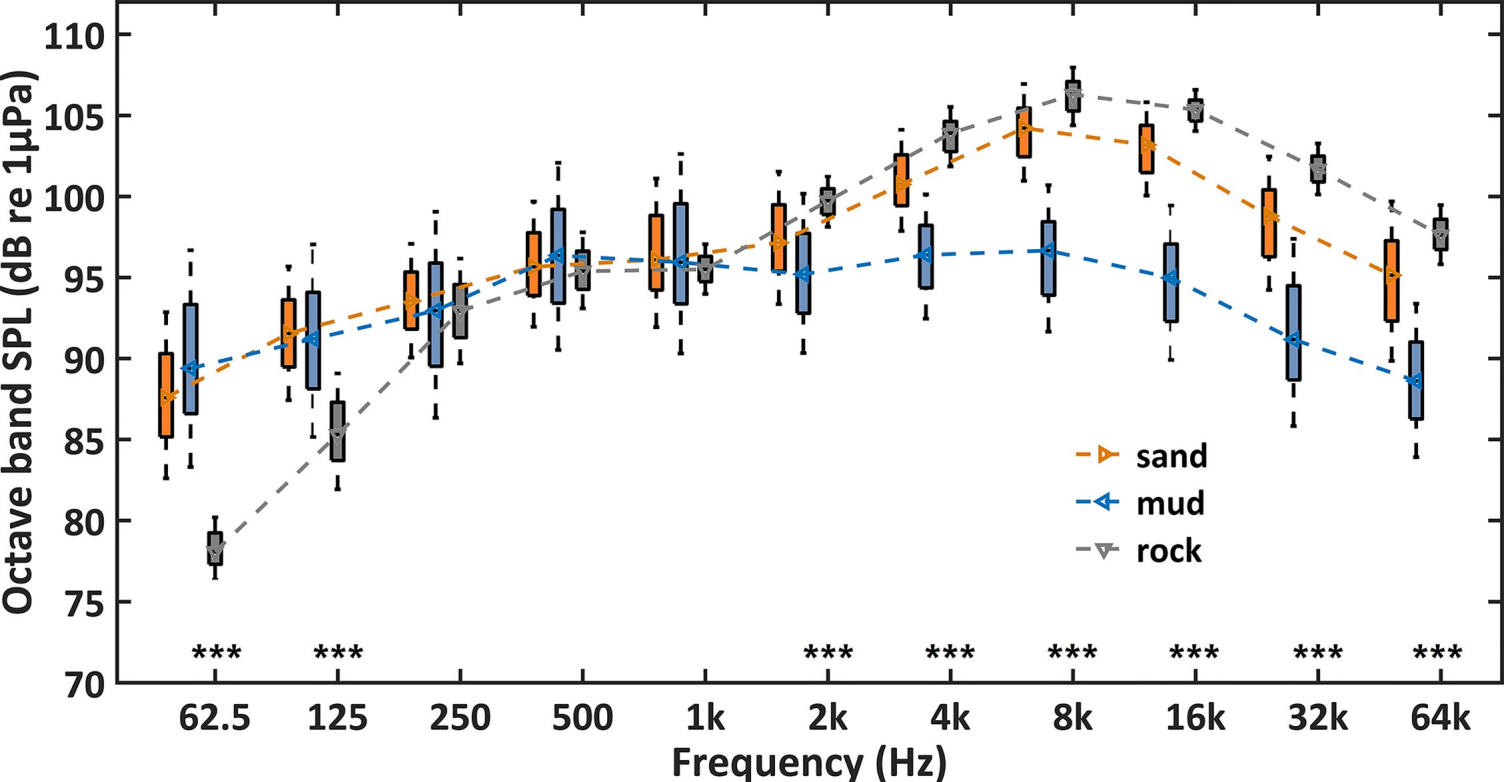
Comparison of octave band sound pressure levels (octave band SPLs) among different seabed environments. The three categories were muddy, sandy and rocky sea floors. Differences are marked with *** for p-level < 0.001 (Kruskal-Wallis test).

Fig 4 showed the comparison of broadband SPLs among sandy, muddy and rocky sea bottoms. Average broadband SPLs for the three seabed environments were 115.4, 112.6 and 114.4 dB re 1μPa, respectively. Besides, the 45th to 55th percentile of broadband SPLs was 112.3 to 117.4 dB re 1μPa for sandy seabed and 109.6 to 115 dB re 1μPa for muddy areas. The smallest variation of broadband SPLs (113.0–114.7 dB re 1μPa) was recorded in the rocky sea bottom (Fig 4). The three seabed categories (sandy, muddy and rocky) were different from each other in terms of broadband SPLs (Mann-Whitney U test, df = 1, p < 0.001). Moreover, these three underwater environments were further compared in terms of octave band SPLs (Fig 5). Octave band SPLs centered at 62.5 and 125 Hz were lower for rocky seabed compare to the other categories (Mann-Whitney U test, df = 1, p < 0.001). Octave band SPLs centered at 2, 4, 8, 16, 32, and 64 kHz were higher for rocky and sandy areas compare to muddy seabed locations (Mann-Whitney U test, df = 1, p < 0.001). Octave band SPLs centered at 250 Hz, 500 Hz, and 1 kHz were similar for the three categories (Kruskal-Wallis test, df = 2, p > 0.05) (Fig 5).

### Temporal patterns

**Fig 6:**
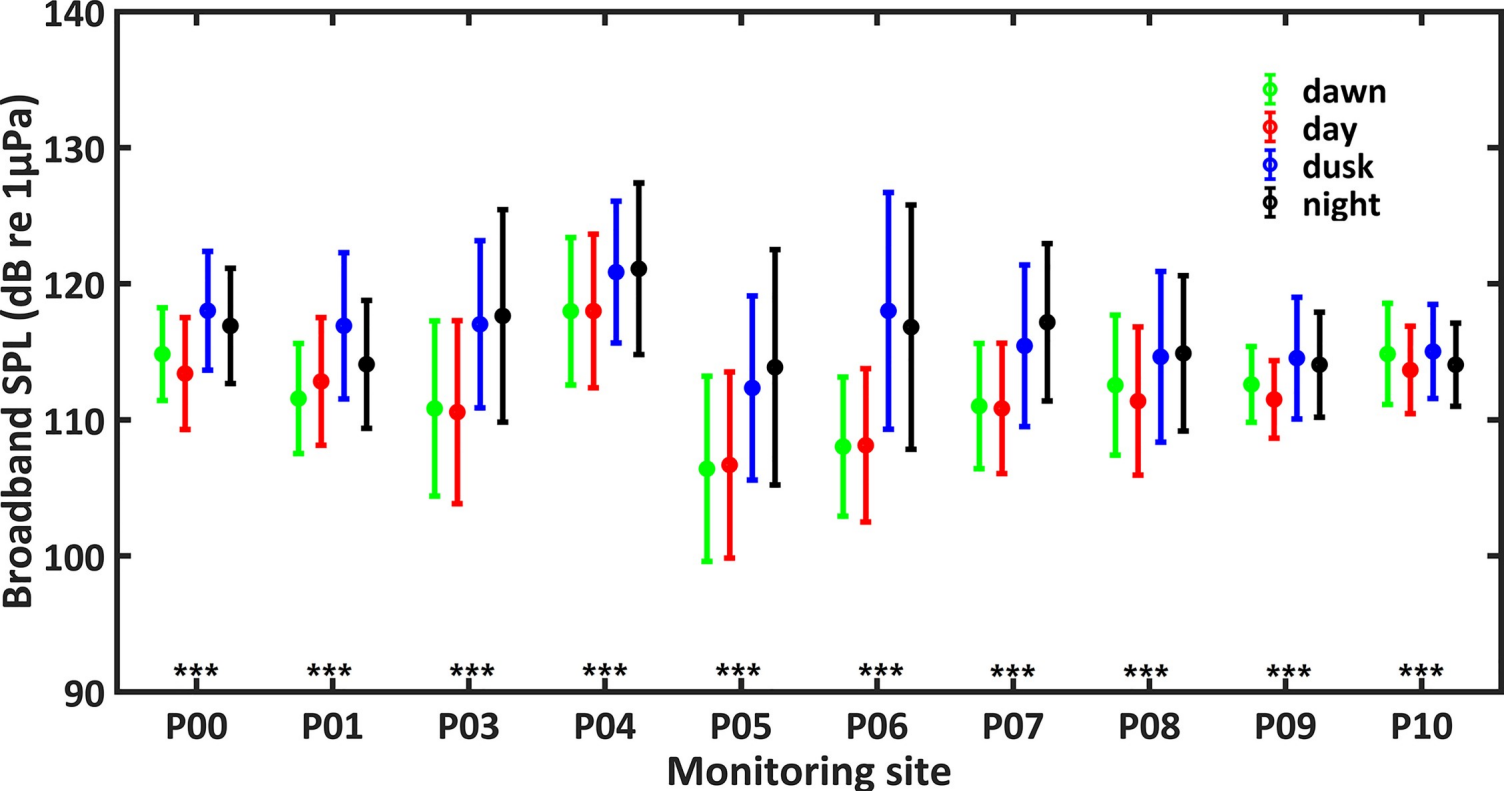
Daily trends of broadband sound pressure levels (broadband SPLs) during one year at the PAM sites. Differences among dawn, day, dusk and night are marked with *** for p-level < 0.001 (Kruskal-Wallis test).

**Fig 7:**
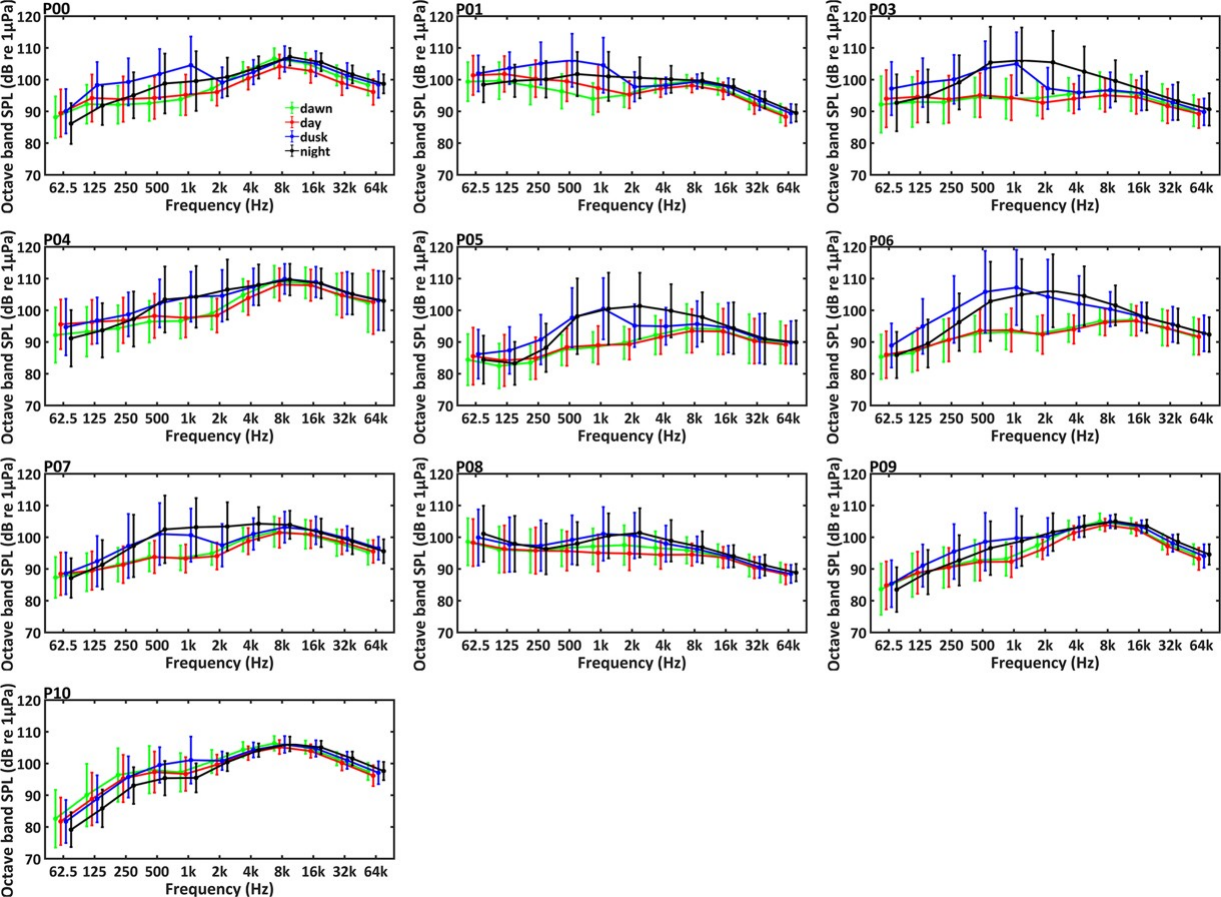
Daily trends of octave band sound pressure levels (octave band SPLs) for different frequency bands during one year at the PAM sites. Differences among dawn, day, dusk and night are marked with *** for p-level < 0.001; ** for p-level < 0.005; * for p-level < 0.05 (Kruskal-Wallis test).

**Fig 8:**
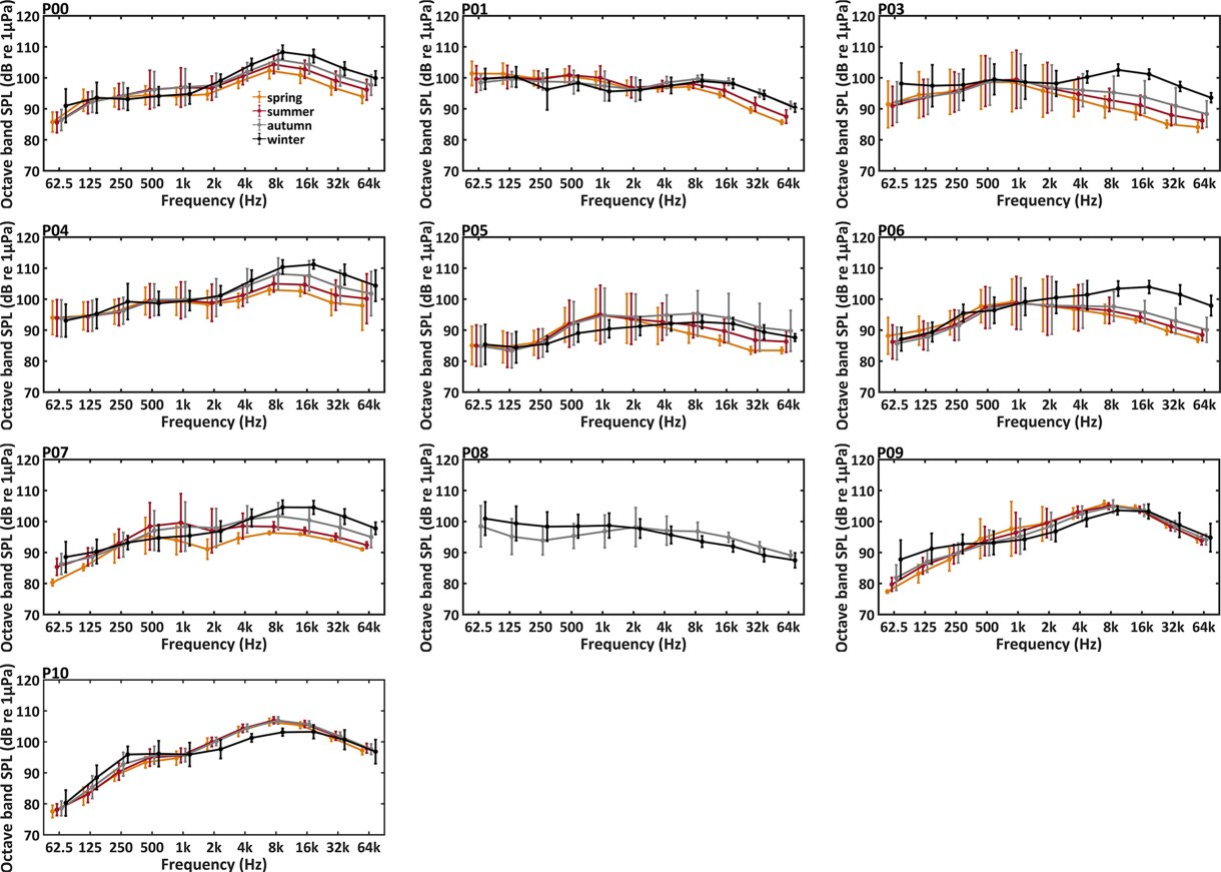
Seasonal trends of octave band sound pressure levels (octave band SPLs) at the PAM sites. Differences among spring (March, April and May), summer (June, July, and August), autumn (September, October and November) and winter (December, January, and February) are marked with *** for p-level < 0.001; ** for p-level < 0.005; * for p-level < 0.05 (Kruskal-Wallis test).

Significant daily and seasonal differences in soundscape were observed at all PAM sites (Figs 6–8).

Daily trends of broadband SPLs at ten sites are shown in Fig 6 and S1 Table. For all PAM sites, except for P10, broadband SPLs were higher during dusk and night than the other times of the day (Mann-Whitney U test, df = 1, p < 0.001). Fig 7 and S2 Table showed the variations of octave band SPLs among time of day. The sites P00, P01, P03, P04, P05, P07, and P09 showed higher octave band SPLs centered at 62.5 Hz during day and dusk than other times (Mann-Whitney U test, df = 1, p < 0.001). All monitoring sites, except P10, showed higher octave band SPLs centered at 500 and 1kHz during dusk and night (Mann-Whitney U test, df = 1, p < 0.001). The site P10 showed highest octave band SPLs centered at 500 and 1 kHz during dusk (Mann-Whitney U test, df = 1, p < 0.001). The sites P00, P01, P03, P05, P08, and P09 showed higher octave band SPLs centered at 2–64 kHz at night compare to the other times of the day (Mann-Whitney U test, df = 1, p < 0.001). Octave band SPLs centered at 2–64 kHz were higher during dusk and night at sites P04, P06 and P07 (Mann-Whitney U test, df = 1, p < 0.001).

Fig 8 and S2 Table showed the seasonal variations of soundscape at each site. The site P01 presented the highest octave band SPLs at 500 and 1kHz during spring and summer (Mann-Whitney U test, df = 1, p < 0.001). At sites P09 and P10, octave band SPLs centered at 62.5, 125 and 250 Hz were higher in autumn and winter compared to the other seasons (Mann-Whitney U test, df = 1, p < 0.001). At sites P00, P01, P03, P04, P05, P06 and P07, octave band SPLs centered at 8, 16, 32 and 64 kHz were higher in autumn and winter (Mann-Whitney U test, df = 1, p < 0.001). At site P09, octave band SPLs centered at 32 and 64 kHz showed no significant seasonal variations (Kruskal-Wallis test, df = 3, p > 0.05). At site P10, octave band SPLs centered at 8 and 16 kHz were lowest in winter.

### Main biological and anthropogenic sound sources

**Fig 9:**
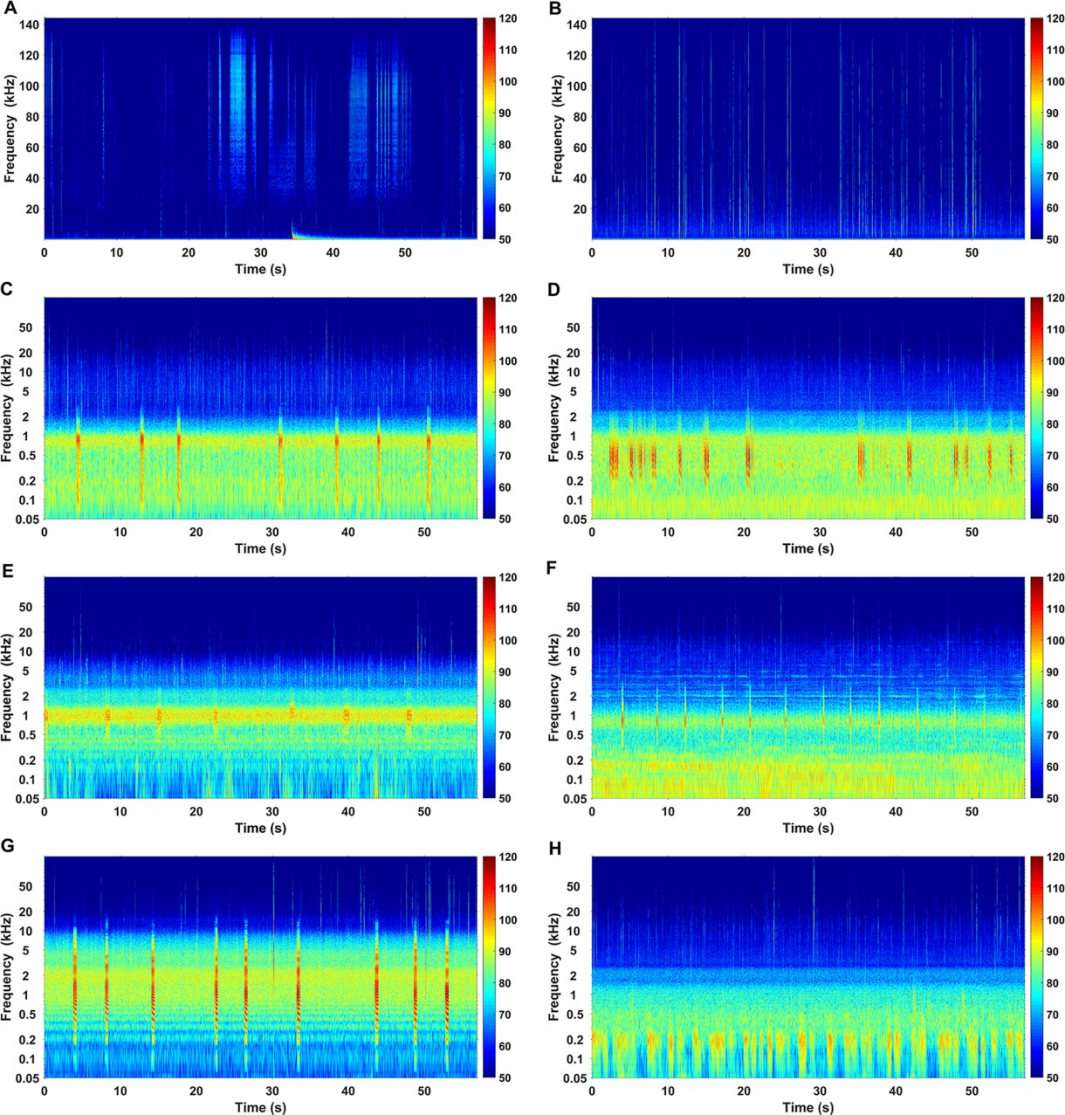
Spectrograms of the selected biological sounds. Spectrograms of “clicks” produced by IPHD (A), the sounds produced by snapping shrimp (B) and six different fish call types (C–H). (FFT length = 144000 points, Hanning window and 50% overlap, the color-bar shows the power spectral density (dB re 1 μPa^2^Hz^-1^)).

**Fig 10:**
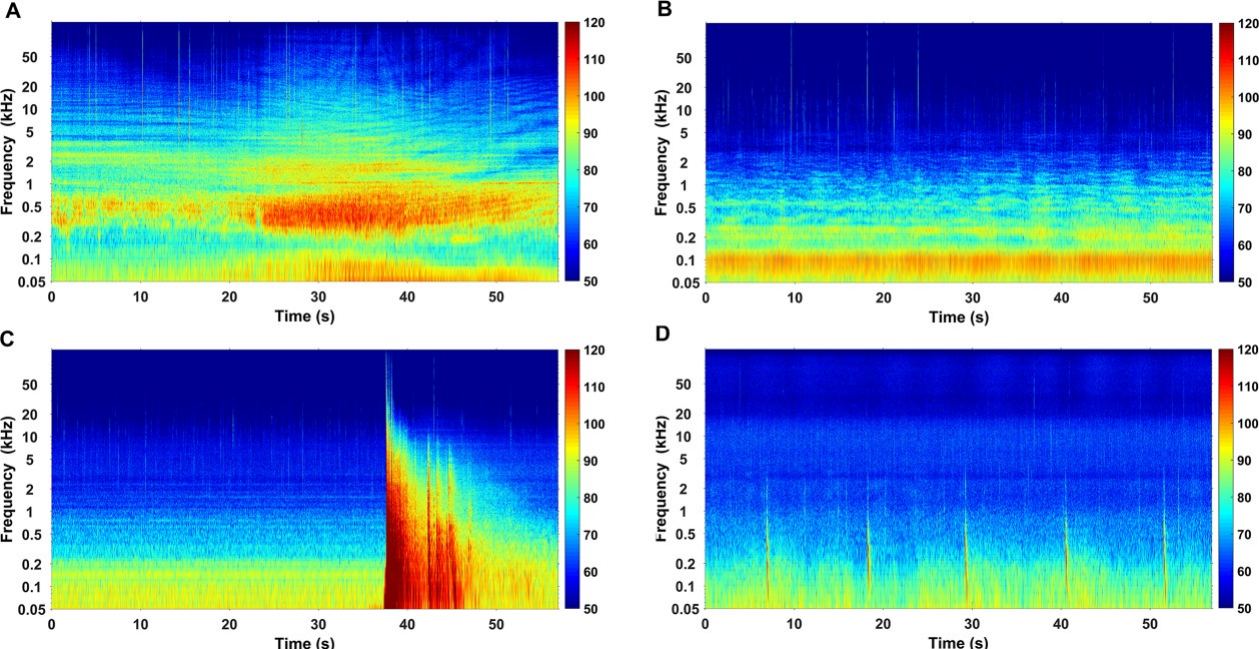
Spectrograms of the selected anthropogenic sounds. Spectrograms of ship noise (A-B), the sound from explosion used for illegal fishing activity (C) and the sound of pile-driving activity (D). (FFT length = 144000 points, Hanning window and 50% overlap, the color-bar shows the power spectral density (dB re 1 μPa^2^ Hz^-1^)).

Prominent biological and anthropogenic sounds recorded in the study area were produced by snapping shrimps, fishes, dolphins, vessel traffic, pile-driving activities, and explosions (Figs 9 and 10).

**Fig 11:**
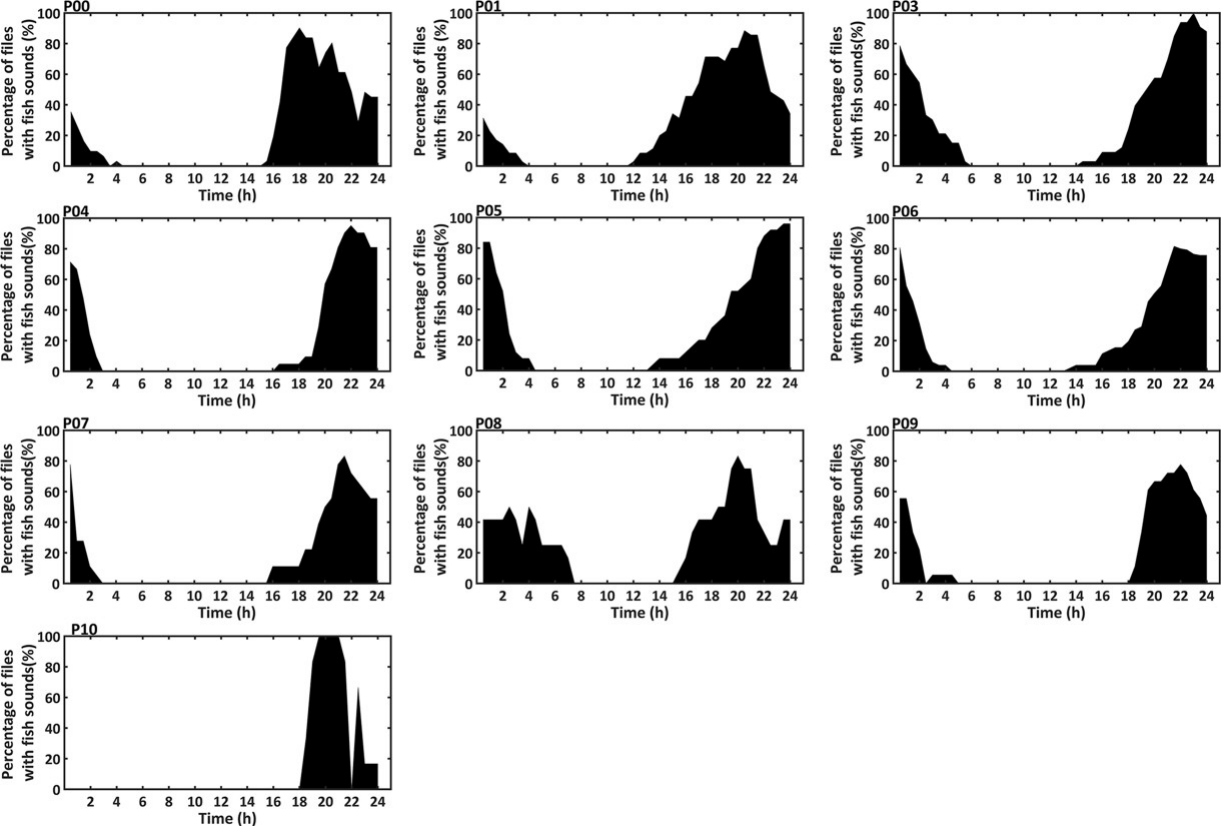
The percentage of files with fish calls in the time of day for the PAM sites. For each site, the percentage of files that contained fish calls were documented at different time of the day by analyzing the spectrogram of three-day sound recordings during new moon period.

Snapping shrimp “snaps” were observed constantly in each recording file at all sites, with frequencies above 2 kHz (Fig 9B). Dolphin “clicks” were sporadically recorded in frequencies above 20 kHz (Fig 9A), confirming that dolphin sounds are a minor component in the variation of the soundscape in Hainan waters [47, 48]. Species-specific identification of the sounds produced by dolphins was not applied in this work. We supposed that the majority of these sounds were produced by IPHD, considering that IPHD is the only dominant dolphin species recorded during our long-term periodical boat-based visual surveys (started in 2013) and local ecological knowledge (LEK) survey in the investigated waters [62], with other dolphin species (i.e., the Indo-Pacific bottlenose dolphin) emitting similar sounds of IPHD were sighted only once, which was in the northernmost part of the investigated waters. Besides, up to six types of sounds from soniferous fishes were found, on which the species-specific identification was not applied. The majority energy of fish sounds was from 0.1 kHz to 2 kHz except one category with a wide frequency band (0.2–10 kHz) (Fig 9C–9H). The percentage of files with fish calls was 24.5%, which were mainly found during the time intervals 17:00–06:00 (Fig 11).

**Fig 12:**
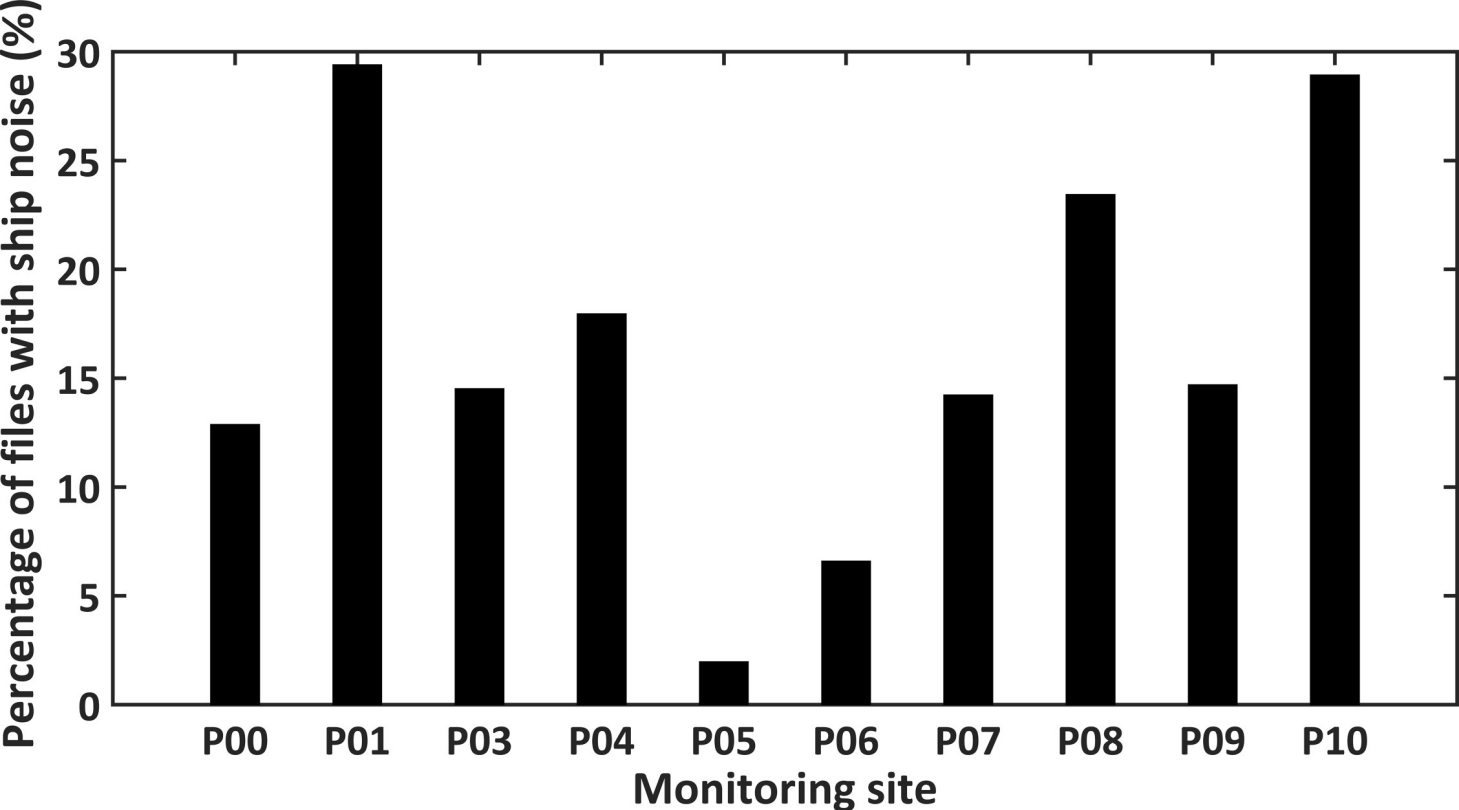
Percentage of files with ship noise. For each site, number of files with ship noise were documented by examining the spectrogram of three-day recordings during new moon period.

Three different man-made sound sources including vessel traffic, explosions and pile-driving activities were identified (Fig 10). The majority of energy for ship noise was below 0.5 kHz (Fig 10B). However, the peak frequency reached 20 kHz with nearby passing vessels or idling engines (Fig 10A) [54]. Percentages of files containing ship noise for the files manually inspected varied from 1.97% to 29.4% among the ten PAM sites (Fig 12). The least number of ship sounds was recorded at site P05 (Fig 12), confirming previous results in the study area [47]. Sounds from explosions produced by illegal fishing activity were found in the 20 Hz–20 kHz range (Fig 10C). Pile-driving pulses were recorded as a series of sharp pulses every few seconds, with the frequency between 50 Hz–1 kHz (Fig 10D) [26]. Few recordings with explosions and pile-driving sounds were reported during the spectrogram analysis of the subsample dataset.

## Discussion

Numerous studies have shown that underwater ambient sound varies in time and space among different marine habitats [3, 63]. However, there is a lack of research on soundscape variation over a long period and a large spatial scale in tropical underwater environments. In the present study, we described the acoustic environment of a tropical habitat of IPHD during one-year monitoring and at ten sampling sites, spanning ~200 km of the coastline.

The monitoring sites P04 and P10 were found to have the highest octave band SPLs centered at 16, 32, and 64 kHz. This could be mainly contributed by biological sounds. Snapping shrimps were considered to be the major source of biological sounds, which were identified in each inspected audio file in this study, same as the previous work documented in other temperate and tropical waters [20, 21]. The typical spectrum of a “snap” has a low-frequency peak between 2 and 5 kHz and energy extending out to 200 kHz [21]. Dolphins also emit high-frequency “clicks” for echolocation and navigation. The observed “clicks” were considered to be mainly from IPHD in this study, while further investigation to classify the sounds from other possible species that emit similar sounds is deserved. However, previous studies [48] claimed that the presence of dolphin clicks has a very little influence on the variation of the soundscape in the investigated waters. Therefore, the higher SPLs at frequencies above 16 kHz for the sites P04 and P10 may reflect the prevalence of snapping shrimp at these two sites. An earlier study [47] also reported that high levels of noise at mid-high frequency bands during the first two months of the monitoring at the site P04 reported were noted, which were observed to be strongly influenced by anthropogenic sounds and natural abiotic phenomena (e.g. tides). The site P05 displayed the lowest broadband SPL and octave band SPLs centered at 125, 250, and 500 Hz, which may due to the least human activities at this site, as previously reported with recordings from the first two months of monitoring [48]. The percentage of files containing ship noise at site P05 was only 1.97%, which is the lowest record of ship activities among the ten sites.

Seabed structure can affect ambient sound in shallow waters [20, 51]. The propagation of sound is greatly influenced by environmental factors, such as water depth [63] and seabed structure [51, 63, 64]. In this study, the ten sites were located at 10–16 m water depth. Only frequencies below 110 Hz might have been affected by normal mode 1 cutoff frequency [63, 65, 66]. Therefore, it is reasonable to consider that the sound levels at frequencies more than 110 Hz for the three different seabed structures (sandy, muddy and rocky seabed) in this study could be compared. The lowest octave band SPLs centered at 125 Hz were recorded for rocky area, which is probably due to the weak water flow and wind-dependent surface waves [27]. Higher octave band SPLs centered at 2, 4, 8, 16, 32 and 64 kHz were observed for rocky and sandy sea bottoms than for muddy area. This was probably related to the fact that attenuation of high-frequency signals is smaller in waters with rocky and sandy bottoms and/or snapping shrimps prefer to dwell in habitat with hard seabed structures. A previous study showed that sandy seabed refracts sounds with a high impedance ratio [63]. In general, a significant correlation between bottom hardness and the number of snaps (shrimps) was reported in coastal marine environments [55]. However, in our PAM survey there was only one monitoring site with rocky bottom, thus further investigations are needed to accurately compare the soundscape of locations with different seabed environments.

Additionally, there was considerable temporal variation of soundscape at each site. The daily patterns of broadband SPLs at the PAM sites were significant, with higher values during dusk and night than the other times of the day at all sites except for P10. The same pattern was identified for octave band SPLs centered at 0.5 and 1 kHz at all sites except P10, which may be a result of evening fish chorus. In this study, fish calls were mainly detected during 17:00–06:00 with most energy levels falling below 2 kHz. Previous studies both in the present investigated area and other waters around the world showed similar daily pattern in acoustic activity of soniferous fishes [49, 50, 67, 68]. Besides, studies in other temperate habitats of IPHD, such as the west coast of Taiwan [49] and the Pearl River Estuary waters [50], reported similar results. In the west coast of Taiwan, the SPLs from 1.2 to 2.4 kHz became higher after sunset, which was associated with the acoustic activity of croakers (family: Sciaenidae) [49]. Also, in the Pearl River Estuary waters, the SPLs in the frequency band between 0.2 and 2.2 kHz were significantly higher during nighttime [50]. In this study, octave band SPLs centered at 2, 4, 6, 8, 16, 32 and 64 kHz during nighttime were higher than other times of day at sites P00, P01, P03, P05, P08 and P09, which may be due to the daily variation of snapping shrimps sounds. Similarly, a study of Lampedusa Island (Mediterranean Sea) found higher octave band SPLs at frequencies from 2 to 64 kHz at night [14]. The acoustic activity of snapping shrimps is influenced by light intensity and time of the day [22, 24]. However, the sites P04, P06, and P07 showed higher octave band SPLs centered at 2–64 kHz during dusk and night than the other times of the day, and the site P10 showed highest octave band SPLs centered at 2–8 kHz during dawn. Possible explanations include different species, habitats and social contexts of snapping shrimps [69].

Seasonal variation of octave band SPLs centered at 8, 16, 32 and 64 kHz were found at sites P00, P01, P03, P04, P05, P06 and P07, with higher values in autumn and winter. Those frequency bands were mainly occupied by impulsive signals of snapping shrimps. As previously reported, the acoustic activity of snapping shrimps was connected to different factors, such as dissolved oxygen concentration [70], water temperature [14] and social context [24]. Higher sound levels at frequencies from 2 kHz to 64 kHz during the summer were reported in coastal waters of the Mediterranean Sea [14], which is different from those in the present study in waters southwest of Hainan Island. This may be due to less variation of water temperature in tropical areas. However, we cannot exclude that the differences found among these different regions could be also related to other environmental and species-specific factors. For the site P08, this study failed to compare octave band SPLs centered at 8, 16, 32 and 64 kHz among spring, summer, autumn and winter due to missing acoustic data during spring and summer. Sites P09 and P10 showed a small seasonal variation in octave band SPLs at 8–64 kHz compared with other sites, which may be due to different species composition and/or environmental conditions influencing the distribution and acoustic activities of snapping shrimps [71].

It is important to note that the waters southwest of Hainan Island were also affected by many anthropogenic activities, including vessel traffic, explosions used for illegal fishing and pile-driving activities. In our study, most of the ship noise had frequencies below 500 Hz, while the ship noise with frequency even up to 20 kHz were also recorded. Such difference was probably related to the differences in ship type/speed and distance from the receiver [54]. The major ship activity in the investigated area was considered to be related to fishing, following these considerations: (1) the China fishery statistics yearbook for 2018 reported that there were over 25,000 motor fishing boats in Hainan and the motor power of more than 80% of the fishing boats was below 44.1kW [72]; (2) the highest number of ships observed in coastal waters southwest of Hainan Island during our periodical visual surveys were fishing boats. The presence of sounds from both explosions for illegal fishing and pile-driving activities were documented in this study, while there were not many of them being detected, which could be due to the manual check of only a subsample of the large dataset. Further investigations are needed to disclose how dolphins respond to these human activities in the study area. Many studies have shown negative effects of these intense anthropogenic noises on marine mammals [9, 37, 39, 41, 73]. Regarding IPHD, studies have shown that the behavioral state of this species can be disrupted by vessel noise [50, 74, 75], and the dolphin sightings in certain waters of the Pearl River Estuary were diminished in areas of high vessel activity [76, 77]. Although IPHD showed a degree of tolerance for vessels due to foraging pressure [60], intense noises from pile driving activities can surely induce behavioral disturbances on marine mammals [78] and cause auditory masking in IPHD whistles [79]. Explosions from illegal fishing activities do acoustically pollute the waters and severely affect the local marine environment. When the animals are close to the explosion site, their survival may be badly affected [80]. Therefore, we strongly recommend the policy makers to strengthen the supervision and regulation of illegal fishing activities in coastal waters southwest of Hainan Island.

Future research should focus on whether human activities have negative impacts on IPHD in waters southwest of Hainan Island. Attention should be specially paid to their physical behavior (such as swim speed, activity state, and movement patterns) and acoustical behavior (such as changes in the intensity, repetition and duration of their sounds). Besides, PAM studies should continue to investigate the occurrence and habitat use of IPHD and quantify the influence of different sound sources on IPHD occurrence. An earlier primary investigation showed that the habitat use of IPHD is strongly related to the presence of soniferous fishes and quiet noise environment in mid-high frequency band [47]. PAM studies could be beneficial for the establishment of conservation actions and policies for the protection of IPHD in coastal waters southwest of Hainan Island.

## Conclusions

The waters southwest of Hainan Island were highly variable in their soundscape characteristics. This one-year PAM study revealed significant spatial and temporal variation in broadband SPLs and octave band SPLs in this region. The sites P04 was nosier than the other sites, and the site P05 was the quietest location in the area. The seabed structure affected the ambient sound. Sandy bottom and rocky bottom had higher broadband SPLs and octave band SPLs centered at 2, 4, 8, 16, 32 and 64 kHz. The PAM sites also showed daily and seasonal variation in broadband SPLs and octave band SPLs, with higher octave band SPLs centered at 0.5 and 1 kHz during dusk and night than other times at all sites except for one, and higher octave band SPLs centered at 8, 16, 32, and 64 kHz were observed in autumn and winter at seven sites. Three main categories of biological sounds were described, including six different types of fish sounds, “snap” produced by snapping shrimps, and “clicks” produced by dolphins. Three main sources of anthropogenic sounds were identified, including vessel traffic, explosions for illegal fishing activities and pile-driving pulses. The soundscape variation illustrated the acoustic complexity of the study area. How IPHD effectively navigates this acoustic complex environment, and the influence of natural abiotic, biotic and anthropogenic sound sources on the dolphin ecology needs to be determined during long-term and large-scale PAM programs. Future further studies in the investigated waters would provide more fundamental information on how to mitigate possible negative effects of different sound sources on IPHD in its tropical habitat, the shallow waters southwest of Hainan Island.

## Supporting information

S1 Table Average broadband sound pressure levels (broadband SPLs) of ten PAM sites. Results of broadband SPLs in different time scales including total recording period, dawn, day, dusk, night, spring, summer, autumn and winter for the ten sites.
(PDF)

S2 Table Average octave band sound pressure levels (octave band SPLs) of ten PAM sites. Results of broadband SPLs in different time scales including total recording period, dawn, day, dusk, night, spring, summer, autumn and winter for the ten sites.
(PDF)
