# Supplementary material for: Long-term and large-scale spatiotemporal patterns of soundscape in a tropical habitat of the Indo-Pacific humpback dolphin (Sousa chinensis)
Source: PLoS One. 2020 Aug 12;15(8):e0236938. doi: 10.1371/journal.pone.0236938 (PMC7423153; doi:10.1371/journal.pone.0236938)
Supplement: S1 Table — Results of broadband SPLs in different time scales including total recording period, dawn, day, dusk, night, spring, summer, autumn and winter for the ten sites. (PDF) [file pone.0236938.s001.pdf]

Time scale	Monitoring site									
	P00	P01	P03	P04	P05	P06	P07	P08	P09	P10
total recording period	115.7	113.3	113.9	119.7	112.6	113.3	114.0	113.7	113.0	114.1
dawn	114.8	111.6	110.8	118.0	106.4	108.0	111.0	112.5	112.6	114.8
day	113.4	112.8	110.6	118.0	106.7	108.1	110.8	111.4	111.5	113.7
dusk	118.0	116.9	117.0	120.8	112.3	118.0	115.4	114.6	114.5	115.0
night	116.9	114.1	117.6	121.1	113.9	116.81	117.2	114.9	114.0	114.0
spring	112.4	113.1	111.7	116.2	118.3	112.8	109.6	-	113.6	115.1
summer	116.2	113.7	112.6	119.3	109.4	110.2	111.6	-	113.3	114.7
autumn	116.9	113.4	114.4	122.6	115.0	114.0	115.4	112.7	112.8	114.9
winter	117.3	113.0	116.5	120.7	117.7	116.3	115.5	114.2	112.9	113.2
