# Supplementary material for: Long-term and large-scale spatiotemporal patterns of soundscape in a tropical habitat of the Indo-Pacific humpback dolphin (Sousa chinensis)
Source: PLoS One. 2020 Aug 12;15(8):e0236938. doi: 10.1371/journal.pone.0236938 (PMC7423153; doi:10.1371/journal.pone.0236938)
Supplement: S2 Table — Results of broadband SPLs in different time scales including total recording period, dawn, day, dusk, night, spring, summer, autumn and winter for the ten sites. (PDF) [file pone.0236938.s002.pdf]

Monitoring site	Time scale	Center frequency (Hz)										
		62.5	125	250	500	1k	2k	4k	8k	16k	32k	64k
	total recording period	86.7	92.4	93.9	95.7	96.3	97.2	101.4	105.3	103.8	100.0	97.1
	dawn	88.2	92.3	92.1	92.5	93.8	97.1	102.5	106.6	105.2	101.3	98.3
	day	89.5	94.2	93.8	94.2	95.2	96.1	100.4	104.1	102.8	99.0	96.1
	dusk	90.0	98.3	99.3	101.8	104.6	99.1	102.4	106.5	105.0	101.3	98.5
P00	night	86.2	91.8	95.1	98.8	99.6	100.8	103.8	107.2	105.5	101.6	98.7
	spring	85.7	92.7	93.6	94.3	94.1	94.7	98.6	102.3	100.9	97.0	94.0
	summer	85.6	91.9	94.0	96.2	96.9	96.7	100.5	104.3	102.8	99.1	96.2
	autumn	86.4	92.3	94.2	96.2	97.0	97.6	102.0	105.9	104.3	100.5	97.7
	winter	91.0	93.6	93.1	94.1	94.8	99.1	104.3	108.3	107.0	102.9	99.9
	total recording period	99.8	100.4	98.7	100.0	98.5	96.6	97.4	98.3	96.6	92.3	88.3
	dawn	99.3	99.7	98.0	96.3	93.9	95.1	98.2	99.5	97.8	93.5	89.4
	day	101.4	101.8	100.3	99.5	97.3	95.3	97.2	98.1	96.5	92.2	88.3
	dusk	101.9	103.6	105.1	106.1	104.5	97.7	98.1	99.4	97.8	93.4	89.4
P01	night	98.4	99.6	100.1	101.8	101.0	100.6	100.1	99.7	97.8	93.6	89.5
	spring	101.4	101.3	99.9	100.7	99.3	96.7	96.6	97.2	94.6	89.6	85.7
	summer	99.6	100.2	99.5	100.9	100.0	96.9	97.4	98.1	96.0	91.5	87.5
	autumn	98.5	99.6	98.8	98.6	97.4	96.3	98.5	99.6	98.7	95.0	91.2
	winter	99.7	100.3	96.2	98.3	95.6	96.1	97.5	99.0	98.1	94.5	90.3
	total recording period	92.3	94.2	95.7	98.8	99.1	96.8	95.6	94.6	92.9	89.8	87.6
	dawn	92.2	93.0	92.9	94.4	94.0	94.1	95.4	96.6	95.8	92.8	90.2
	day	94.0	94.6	93.8	95.1	94.3	92.7	94.0	95.1	94.5	91.7	89.2
	dusk	97.2	99.0	100.1	103.5	105.0	97.2	95.9	96.7	95.7	92.8	89.8
P03	night	92.7	94.9	99.1	105.4	106.1	105.5	102.5	99.6	96.5	93.2	90.6
	spring	91.4	94.5	95.5	98.5	98.6	95.8	93.3	90.6	88.6	85.1	84.1
	summer	90.9	93.5	95.4	99.0	99.5	96.8	94.7	92.9	91.2	88.0	86.2
	autumn	92.2	93.9	95.4	98.7	99.0	96.8	96.0	95.3	93.8	91.0	88.3
	winter	98.1	97.4	97.7	99.5	98.6	98.2	100.2	102.5	101.2	97.2	93.6
	total recording period	93.8	94.7	96.5	99.5	99.7	99.8	103.0	106.8	106.6	103.0	101.1
	dawn	92.2	93.3	94.3	96.4	96.6	99.0	104.8	109.0	108.6	105.1	102.3
	day	95.6	96.3	96.8	98.2	97.6	98.4	103.8	108.1	107.9	104.7	102.6
	dusk	94.7	96.8	98.7	102.2	104.3	104.6	107.4	109.9	108.9	105.4	103.1
P04	night	91.2	93.7	97.2	103.4	104.2	106.5	108.0	109.7	108.4	105.1	103.0
	spring	94.0	94.6	95.9	99.4	99.3	98.3	99.5	103.0	102.6	98.9	97.9
	summer	93.9	94.4	95.8	99.5	99.6	98.8	101.2	105.0	104.6	101.3	100.1
	autumn	93.8	94.6	96.3	99.8	99.9	100.5	104.4	108.2	107.6	103.8	101.8
	winter	93.1	95.3	99.2	98.7	99.5	101.2	106.0	110.4	111.2	107.9	104.3
	total recording period	85.0	83.8	85.8	91.6	94.3	93.6	93.3	92.9	91.4	88.4	87.5
	dawn	84.4	82.5	83.5	87.5	88.4	89.7	92.2	94.3	94.0	91.0	89.9
	day	85.5	84.2	84.9	88.4	89.0	89.3	91.5	93.5	93.3	90.3	89.2
	dusk	86.2	87.4	90.8	97.7	100.5	95.1	95.0	95.7	94.5	91.1	89.9
P05	night	84.4	83.3	88.2	98.1	100.5	101.4	99.8	97.9	94.4	91.0	89.9
	spring	85.0	84.6	85.9	91.7	95.0	93.8	91.4	89.0	86.7	83.5	83.4
	summer	85.0	83.6	85.6	92.1	95.0	93.6	92.7	91.5	89.7	86.8	86.3
	autumn	84.8	83.3	85.9	92.2	94.8	94.3	94.9	95.4	94.0	90.9	89.8
	winter	85.4	84.5	85.6	89.0	90.4	91.2	92.1	92.6	92.1	89.4	87.6
	total recording period	86.5	88.5	92.3	97.2	98.8	98.3	98.0	97.7	96.1	93.2	90.3
	dawn	85.3	86.6	90.5	92.6	93.2	92.7	94.4	96.6	97.0	94.6	91.9
	day	86.0	87.7	90.7	93.6	93.8	92.3	94.0	96.2	96.7	94.4	91.6
	dusk	88.9	94.9	100.3	105.8	107.2	104.3	102.1	100.4	98.0	95.6	92.7
P06	night	85.9	89.5	96.3	102.8	105.0	106.1	104.5	101.5	97.7	95.1	92.3
	spring	88.2	89.8	92.4	97.7	99.1	98.0	96.7	95.3	93.3	89.8	87.0
	summer	86.2	88.3	91.6	97.3	98.8	97.9	97.2	96.3	94.2	91.2	88.5
	autumn	85.7	87.8	91.6	97.2	98.5	98.0	97.8	97.6	95.8	93.0	90.1
	winter	87.1	89.4	95.5	96.4	99.2	100.5	101.4	103.4	103.9	101.5	97.9
	total recording period	86.5	89.0	92.8	96.9	98.0	97.4	100.1	101.4	100.4	98.0	94.9
	dawn	87.3	89.0	91.4	93.7	93.5	94.9	99.3	101.8	101.0	98.3	95.2
	day	88.5	89.5	91.3	93.8	93.3	94.1	98.8	101.4	100.9	98.5	95.5
	dusk	88.6	92.4	97.2	101.0	100.7	97.5	101.0	103.2	102.1	99.6	96.5
P07	night	87.1	91.4	97.3	102.5	103.2	103.4	104.3	103.9	101.4	98.7	95.6
	spring	80.3	85.2	91.3	95.4	94.0	91.1	94.6	96.3	95.9	94.0	91.0
	summer	85.4	88.6	92.9	98.4	99.6	97.0	98.5	98.3	97.1	95.1	92.4
	autumn	86.3	88.6	92.5	97.1	98.4	97.9	100.7	101.7	100.4	98.1	95.0
	winter	88.5	90.3	93.2	94.7	95.4	96.9	101.1	104.6	104.5	101.6	97.8
P08	total recording period	99.5	96.9	95.7	96.6	97.6	98.0	96.5	95.5	93.7	90.7	88.4

	dawn	98.6	96.1	95.7	96.6	97.1	97.5	96.7	95.9	94.2	91.1	88.9
	day	98.3	96.3	95.8	95.6	95.1	94.9	94.5	94.5	93.6	90.4	88.3
	dusk	99.9	97.7	97.4	99.2	101.1	100.5	98.0	96.2	93.9	90.5	88.4
	night	101.1	97.9	96.3	98.0	100.4	101.4	98.9	96.8	94.0	91.2	88.8
	spring	-	-	-	-	-	-	-	-	-	-	-
	summer	-	-	-	-	-	-	-	-	-	-	-
	autumn	98.5	95.1	93.8	95.3	96.8	98.2	97.0	96.8	94.9	91.8	89.1
	winter	100.9	99.4	98.4	98.5	98.7	97.7	95.7	93.5	92.0	89.1	87.5
P09	total recording period	82.8	87.8	90.5	93.3	95.3	98.3	102.2	104.5	103.5	98.6	94.3
	dawn	83.6	88.0	90.4	92.7	93.2	97.4	102.4	104.9	103.6	98.6	94.6
	day	84.8	88.8	90.5	92.2	92.3	96.2	101.4	103.9	102.5	97.2	93.2
	dusk	85.3	91.1	95.5	98.6	99.7	100.2	103.2	104.9	103.3	98.1	94.1
	night	83.5	89.0	92.7	96.6	98.8	101.6	103.4	105.0	103.5	98.5	94.6
	spring	77.4	83.1	87.7	94.4	97.6	99.2	102.6	105.6	104.3	99.0	94.4
	summer	79.7	85.7	89.4	93.7	96.4	99.5	103.0	105.0	103.8	98.4	93.9
	autumn	81.9	87.1	89.8	93.1	95.2	98.5	102.5	104.8	103.6	98.5	94.2
	winter	87.8	91.2	92.7	93.1	94.2	96.8	100.8	103.6	103.2	98.8	94.8
P10	total recording period	78.9	85.7	93.1	95.6	95.7	99.3	103.5	105.8	104.9	101.5	97.5
	dawn	82.6	90.0	96.3	98.0	97.2	100.6	104.3	106.3	105.1	101.5	97.5
	day	81.7	88.8	95.3	97.3	96.7	99.7	103.2	105.2	103.9	100.1	96.1
	dusk	81.7	88.9	95.8	99.5	101.1	100.9	104.3	106.0	104.7	101.0	97.1
	night	79.1	85.9	93.1	95.4	95.5	100.4	104.2	106.2	105.1	101.6	97.6
	spring	77.5	82.5	89.0	93.4	94.7	99.3	103.3	106.4	105.4	101.4	97.2
	summer	78.1	83.1	90.4	94.9	95.6	99.9	104.3	106.9	105.7	101.9	97.9
	autumn	78.5	85.4	92.8	95.7	95.7	100.1	104.5	106.9	105.6	101.8	97.6
	winter	80.3	88.5	95.9	96.2	95.9	97.6	101.3	103.1	103.2	100.7	96.8
